# Predictive models for codend size selectivity for four commercially important species in the Mediterranean bottom trawl fishery in spring and summer: Effects of codend type and catch size

**DOI:** 10.1371/journal.pone.0206044

**Published:** 2018-10-22

**Authors:** Jure Brčić, Bent Herrmann, Antonello Sala

**Affiliations:** 1 Department of Marine Studies, University of Split, Split, Croatia; 2 SINTEF Fisheries and Aquaculture, Fishing Gear Technology, North Sea Science Park, Hirtshals, Denmark; 3 University of Tromsø, Tromsø, Norway; 4 Italian National Research Council (CNR), Institute of Biological Resources and Marine Biotechnologies (IRBIM), Ancona, Italy; Fisheries and Oceans Canada, CANADA

## Abstract

Models to predict codend size selectivity for four major commercial species—European hake *(Merluccius merluccius)*, Norway lobster (*Nephrops norvegicus*), deep-water rose shrimp *(Parapenaeus longirostris*), and Atlantic horse mackerel *(Trachurus trachurus*)–in Mediterranean bottom trawl fisheries were established based on data collected during fishing trials using the two legal codends: a 40 mm square-mesh codend and a 50 mm diamond-mesh codend. The models were applied to predict the extent to which size selection depend on codend type, also accounting for the potential effect of codend catch size and fishing season. The size selectivity of the two codends was evaluated and compared in identical simulated controlled conditions. Mesh type significantly affected the size selection of Norway lobster alone, with a slightly better performance of the 40 mm square-mesh codend. A high risk of retention of undersized individuals was predicted for both codends for all species except Norway lobster.

## Introduction

The European Commission has identified high levels of discarding as a major structural weakness of the previous Common Fisheries Policy (CFP) [1]. The new CFP aims to reduce this wasteful practice [2]. In the Mediterranean region, discards have been increasing in the past 70 years [3] and now account for 18.6% of the total catch; bottom trawls are responsible for the bulk of discards [4]. Several measures have been devised to reduce bottom trawl discarding in the EU, among them technological modifications that improve gear selectivity through changes in codend mesh size and/or geometry [5–8]. However, the multispecies nature of Mediterranean bottom trawl fisheries makes it difficult to optimise codend selectivity for all species through changes in mesh size.

In several Mediterranean countries bottom trawl selectivity is currently managed by regulating minimum mesh size [9,10], not mesh type. As a result, diamond-mesh codends are those most widely used in the region. Council Regulation (EC) No. 1967/2006 allowed codends with a minimum mesh size of 40 mm (regardless of mesh type) to be used by EU trawlers fishing in the Mediterranean until June 30^th^ 2008; from July 1^st^ 2008 they have been required to use a 40 mm square-mesh (SM), or "at the duly justified request of the ship-owner", a 50 mm diamond-mesh (DM) [11]. Since the Regulation does not provide a precise definition of “duly justified request”, Member States have freely interpreted the provision. Article 15 of Regulation (EU) No. 1343/2011, which has amended Council Regulation (EC) No. 1967/2006 [12], lays down the minimum trawl codend mesh size to be used in Black Sea fisheries; it requires the earlier 40 mm mesh codends to be replaced with 40 mm SM codends or, at the duly justified request of the ship-owner, with 50 mm DM codends having an acknowledged size selectivity "equivalent to or higher than that of 40 mm square-mesh codends". This has raised the need for comparing the size selectivity of 50 mm DM and 40 mm SM codends for commercially important species in Mediterranean trawl fisheries. However, other factors that may affect size selection should also be considered when comparing the size selectivity of different codends. One such factor is codend catch size, which is often measured in terms of codend catch weight at the end of a haul. Since experimental [13,14] and theoretical [15–17] studies have found that the size selection of DM codends in trawl fisheries can be influenced by the weight of the catch in the codend, this effect should be considered when predicting and comparing the size selection of legal codends in Mediterranean bottom trawl fisheries. An additional factor that may affect codend size selection is season, in relation to differences in water temperature and/or fish condition; for instance, this has been reported for haddock in DM codends [18]. To date, few studies have directly compared the selectivity of 40 mm SM and 50 mm DM codends [19–21] and none have investigated the potential effect of codend catch size and season.

Based on the above considerations, the objective of this study was to establish predictive models for codend size selection of the two legal codends for four major commercial species that are going to be subject to the landing obligation in Mediterranean bottom trawl fisheries—European hake (*Merluccius merluccius*), Norway lobster (*Nephrops norvegicus*), deep-water rose shrimp (*Parapenaeus longirostris*), and Atlantic horse mackerel (*Trachurus trachurus*)—taking into account the effect of codend catch size and fishing season. The size selection performance of the two codends was evaluated under identical and controlled conditions using established predictive models.

## Material and methods

### Ethics statement

This study did not involve endangered or protected species. Experimental fishing was conducted on board a commercial fishing vessel in accordance with the fishing permit granted by the Italian Ministry of Agriculture and Forestry—Fishery and Aquaculture directorate (DG PEMAC 0007137). No other authorization or ethics board approval was required. No information on animal welfare or on steps taken to mitigate fish suffering and methods of sacrifice is provided, since the animals were not exposed to any additional stress other than that involved in commercial fishing practices.

### Experimental design

**Fig 1:**
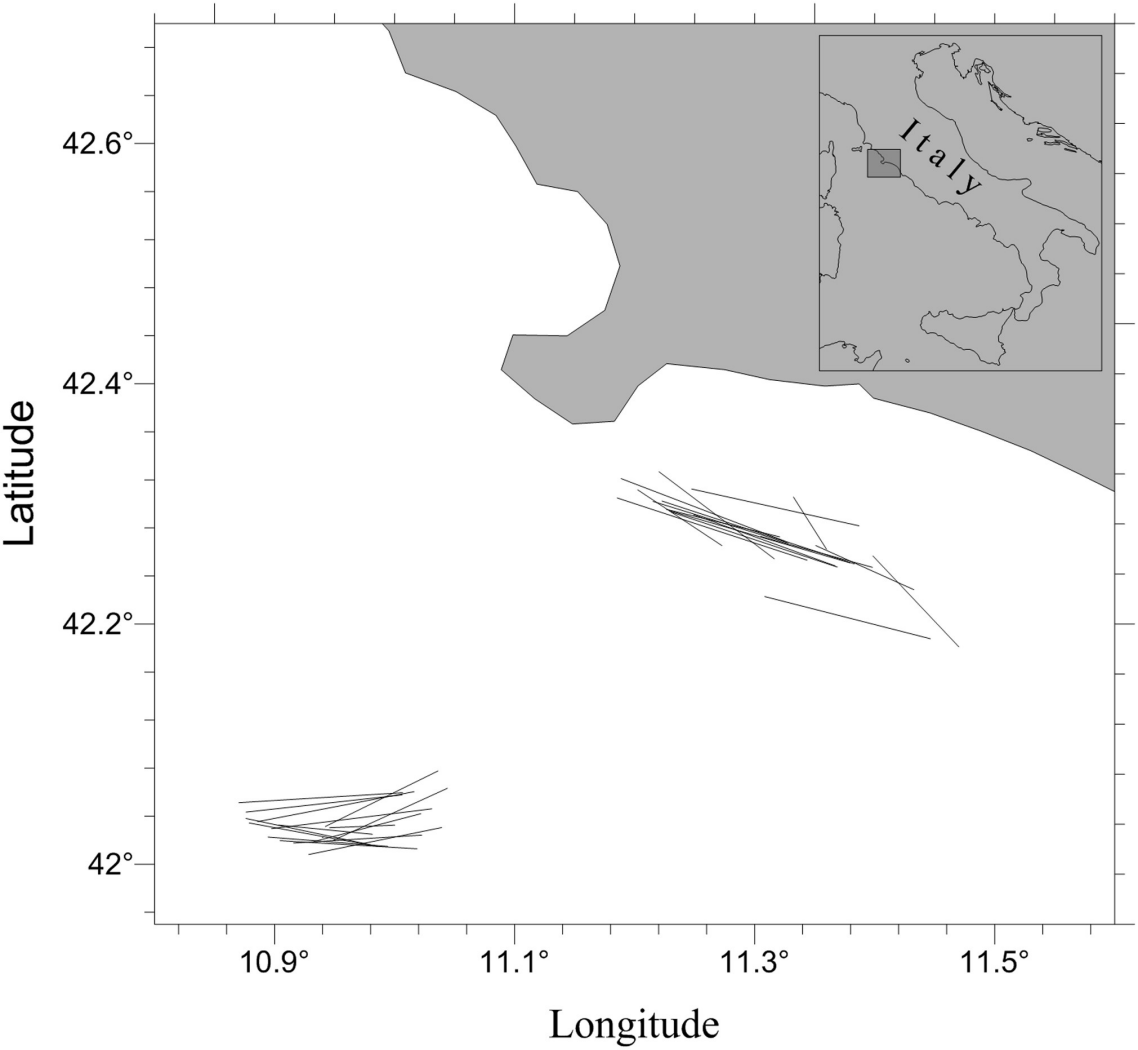
Map of the area where the sea trials were conducted.

Experimental size selection data were collected in spring (March) and summer (July) 2012. Sea trials were performed in the Tyrrhenian Sea (Fig 1) on board the commercial fishing vessel “Angela Madre” (206 kW, LOA 22.7 m, 67 GT), which was equipped with a typical two-face Mediterranean bottom trawl [22].

The gear was made entirely of knotless polyamide (PA) netting; it was 94 m long from the wing tips to the codend and the fishing circle, headline and footrope measured respectively 59.4 m, 45 m, and 55 m. The gear was rigged with Vee type otterboards (1600 x 1000 mm, 190 kg), 230 m long sweeps, and 1600 m long warps, and the rigging was identical to the one commonly used in commercial Tyrrhenian Sea trawl fisheries.

The two codends used in the sea trials were a 5.7 m long (110 mesh) DM codend made of 51.9 ± 0.3 (±SD) mm PA mesh netting with 246 meshes in the circumference (hereinafter DM50 codend) and a 5.5 m long (275 mesh) SM codend made of 40.2 ± 0.65 mm (±SD) PA mesh netting with 140 meshes in the circumference (hereinafter SM40 codend). Codend mesh size was measured with an OMEGA mesh gauge while the netting was wet [23].

The last tapered section of the belly, where the codends were attached, consisted of 44 mm diamond netting with 280 meshes in the circumference. Codend selectivity was estimated using the covered codend method [24]. The codend cover was made of knotless PA netting with a nominal mesh size of 20 mm and was supported by two aluminium hoops to prevent the masking effect [24]. The hoops were used because they are not dependent on the water flow to maintain shape and are therefore preferred if the gear is not too large, as in this case. They were placed respectively 2.5 and 5 m from the point where the cover was attached to the last section of the trawl belly. The circumference of the codend cover was 1.5 times that of the codend [25].

At the end of each haul, the catch found in the codend and codend cover was sorted and weighed separately. The total length (TL) of Atlantic horse mackerel and European hake was measured to the nearest 0.5 cm, whereas the carapace length (CL) of Norway lobster and deep-water rose shrimp was measured to the nearest mm. In case of catches too large, to enable measurement before the arrival on deck of the next haul, some species were subsampled before length measurement.

### Size selectivity data analysis

The size selectivity analysis was carried out separately for each of the four species according to the procedure described below. For each haul, the probability that a fish of length *l* would be retained after entering the codend was modelled with the following logistic curve [24]:

$$r(l,L50,SR)=\frac{\text{exp}((l-L50)\times \text{ln}(9)/SR)}{1+\text{exp}((l-L50)\times \text{ln}(9)/SR)}$$ (1)

where *L50* is the 50% retention length and *SR* is the difference between the 75% retention length and the 25% retention length [24]. The values of *L50* and *SR* were estimated by fitting the logistic curve (1) to the experimental data obtained by recording the length class-dependent retention probability using maximum likelihood estimation [24,26]. The goodness of fit was evaluated based on the p-value [24]. The curve was judged to provide an acceptable description of experimental data if the p-value was > 0.05. A fixed and random effect model, proposed by [27], was used to analyse the data in two steps. In the first step, the *L50* and *SR* values of each haul and their covariance matrix were estimated as described above. In the second step, which took into account both the uncertainty in the individual hauls and between-haul variation in size selection, the results were combined over hauls to predict mean *L50* (*L50*_*mean*_) and mean *SR* (*SR*_*mean*_). This step considered the potential fixed effect of codend design: *DM50* (0 for the 40 mm SM codend, 1 for the 50 mm DM codend); *CATCH* (total codend catch weight at the end of each haul); and *SEASON* (0 = spring and 1 = summer). All other uncontrolled/unmeasured factors on haul level were considered as random effects. The resulting model was as follows:

$$\begin{array}{c} {L50}_{mean}=a_{0}+a_{1}\times DM50+a_{2}\times CATCH+a_{3}\times SEASON+a_{4}\times DM50\times CATCH+a_{5}\times DM50\times SEASON\\ {SR}_{mean}=b_{0}+b_{1}\times DM50+b_{2}\times CATCH+b_{3}\times SEASON+b_{4}\times DM50\times CATCH+b_{5}\times DM50\times SEASON \end{array}$$ (2)

In (2), *a*_*0*_ and *b*_*0*_ are the intercept values for *L50* and *SR* using the SM40 codend, considered as the baseline design; *a*_*1*_ and *b*_*1*_ quantify the effect of switching from SM40 to DM50; *a*_*2*_ and *b*_*2*_ are the effects of total codend catch weight at the end of the haul; *a*_*3*_ and *b*_*3*_ are the effects of season on selectivity; *a*_*4*_ and *b*_*4*_ model the interactions between *DM50* and *CATCH* on *L50* and *SR*, respectively; finally, *a*_*5*_ and *b*_*5*_ model the interaction between *DM50* and *SEASON*.

We also considered all possible sub-models that could be derived from model (2), by removing one or more terms at a time, obtaining a total number of 4096 candidate models for (*L50*_*mean*_, *SR*_*mean*_). Predictive models for codend size selectivity are often established by choosing the one with the lowest AIC value [28,29] or, alternatively, through successive elimination of insignificant parameters [30,31]; however, both approaches require one model to stand clearly out as the model of choice, which often does not happen. To overcome this problem, we decided to consider all the models that showed some likeliness of being the model of choice for the dataset using a technique known as multi-model inference or model averaging [32]. Briefly, this approach makes predictions using a weighted average, where several models are weighted according to how likely they are compared to each other, thus obviating the need for selecting a single model as the best one to make predictions. The 4096 candidate models were ranked and weighted according to their AICc values [32], which are calculated as AIC with a correction for finite sample sizes in the data. Models showing AICc values within +10 of the value of the model with the lowest AICc value (*AICc*_*min*_) were considered for the estimation of *L50*_*mean*_ and *SR*_*mean*_ according to the procedure described by [33] and [34]. Hereinafter, “predictive model” is the term used for the result of this multi-model averaging, which was calculated as follows:

$$\begin{array}{c} ({L50}_{mean},{SR}_{mean})=\sum_{i}w_{i}\times {\left({L50}_{mean},{SR}_{mean}\right)}_{i}\\ with\\ w_{i}=\frac{exp(0.5\times (AICc_{i}-AICc_{min}))}{\sum_{j}exp(0.5\times (AICc_{j}-AICc_{min}))} \end{array}$$ (3)

and where the summations are over the models with an *AICc* value within +10 of *AICc*_*min*_. The subscripts *i* and *j* refer to the candidate models. *w*_*i*_ indicates the Akaike weights, which quantify the contribution of each model considered in the predictive model.

The *L50* and *SR* data of each haul and their 95% confidence intervals (CI) were plotted against the codend catch weight of both seasons together with the estimations obtained by applying the predictive model with CI; both between-haul variation and model uncertainty were considered to establish whether the predictive model represented the experimental individual haul results with sufficient accuracy as described in [29,35].

### Prediction of the performance of the SM40 and the DM50 codend

The size selection properties of the two legal codends were evaluated and compared in identical simulated controlled conditions. The size selectivity of each codend was predicted separately for spring and summer (data were not collected in autumn or winter) based on codend catch weights of 50 kg and 100 kg, respectively, using the predictive models developed as described above. The codend catch weights of 50 kg and 100 kg were selected because they were in the range of most of the experimental hauls. These factors provided four simulated scenarios in which the size selection properties of the two codends were compared. For each scenario, the predicted size selection curves of the SM40 and the DM50 codend were plotted together, to establish whether their 95% CI overlapped. Overlap indicated that the size selectivity of the two codends in the relevant scenario was not significantly different.

Since the predicted size selectivity is independent of population size structure, the exploitation pattern indicators [8,29,36] were also calculated. These indicators depend directly on the population size structure encountered by the gear and provided additional information for the evaluation of the catch performance of each codend. Their values were calculated using the size selection predictions made for each simulated scenario and the population size structure of each species caught during the experimental hauls. This allowed simulating the population structure retained by the codend and the codend cover when the gear encountered a certain population. The simulated catch was then used to calculate the following exploitation pattern indicators:

$$\begin{array}{l} nP-=100\times \frac{\sum_{l<MCRS}NT_{l}}{\sum_{l<MCRS}\left(NT_{l}+NC_{l}\right)}\\ nP+=100\times \frac{\sum_{l>MCRS}NT_{jl}}{\sum_{l>MCRS}\left(NT_{l}+NC_{l}\right)}\\ nRatio+=\frac{\sum_{l<MCRS}NT_{l}}{\sum_{l>MCRS}NT_{l}}\\ dnRatio=100\times \frac{\sum_{l<MCRS}NT_{l}}{\sum_{l}NT_{l}} \end{array}$$ (4)

where *NT*_*l*_ and *NC*_*l*_ are the number of individuals of length *l* retained by the codend and cover, respectively.

*nP-* and *nP+* are respectively the percentage of retained individuals below and above the Minimum Conservation Reference Size (MCRS), taking into account the size structure of the population encountered during the trials. An *nP-* value close to 0 and an *nP+* value close to 100 would be preferable. *nRatio* is the number of retained individuals under the MCRS to each retained individual above the MCRS. The *dnRatio* is the percentage of individuals under the MCRS retained in the codend. *nRatio* and *dnRatio* should be as low as possible.

The uncertainty in the indicator values for each species was calculated based on the uncertainty in the predicted size selection curves using SELNET software [28,36–38]. The plots were made with R software [39] using the “ggplot2” package [40].

## Results

### Establishment of the predictive models

A total number of 32 valid hauls were carried out with the two codends. The parameters used for modelling are reported in Table 1.

**Table 1:** Parameters used for modelling.

Haul	Season	DM50	Catch [kg]
1	0	1	44.71
2	0	1	52.99
3	0	1	57.85
4	0	1	56.04
5	0	1	112.41
6	0	1	97.65
7	0	1	46.25
8	0	1	57.22
9	0	0	55.89
10	0	0	68.70
11	0	0	65.71
12	0	0	51.98
13	0	0	102.68
14	0	0	137.21
15	0	0	85.31
16	0	0	32.40
17	1	0	92.03
18	1	0	80.99
19	1	0	69.96
20	1	0	42.25
21	1	0	51.00
22	1	0	49.81
23	1	0	34.48
24	1	0	52.45
25	1	1	88.19
26	1	1	55.54
27	1	1	71.88
28	1	1	71.22
29	1	1	41.01
30	1	1	40.58
31	1	1	37.72
32	1	1	43.28

Haul: Haul ID; Season: categorical variable where 0 = March and 1 = July; DM50: categorical variable where 0 = 40 mm square-mesh codend and 1 = 50 mm diamond-mesh codend; Catch [kg]: continuous variable representing the total codend catch weight at the end of each haul.

#### Atlantic horse mackerel

The predictive model for Atlantic horse mackerel was obtained using data from 12 hauls, for which it was possible to obtain a size selection curve. Overall, 458 individuals were caught with the SM40 codend (208 length measured) and 1213 with the DM50 codend (767 length measured) (Table 2). Fitting the logit curve (1) to the data from each considered haul consistently yielded p-values > 0.05, indicating that the curve effectively described the experimental size selection data of all hauls (Table 2). The results from each of the 12 hauls were then used to identify the predictive model for *L50* and *SR*. Four models produced an AICc value within +10 of the model with the lowest value (Table 3).

**Table 2:** Estimated selection parameters and fit statistics for Atlantic horse mackerel (12 hauls).

Haul	NT	qNT	NC	qNC	L50 [cm] (±CI)	SR [cm] (±CI)	p-value	Deviance	DOF
1	65	1.000	78	1.000	16.30 (±0.72)	3.82 (±1.60)	0.2998	21.69	19
2	108	1.000	9	1.000	15.25 (±1.62)	2.87 (±1.79)	0.9895	14.34	29
3	160	1.000	14	1.000	14.48 (±1.25)	2.88 (±1.51)	0.9980	15.02	34
4	153	0.500	34	1.000	11.76 (±2.99)	5.55 (±3.07)	0.8360	13.05	19
9	46	1.000	5	1.000	11.84 (±3.49)	3.32 (±3.89)	0.9964	4.87	16
10	43	0.250	2	1.000	14.45 (±13.79)	0.10 (±3.03)	1.0000	0.00	20
12	60	0.500	1	0.500	14.25 (±252.83)	0.10 (±35.96)	1.0000	0.00	22
21	22	1.000	10	0.200	11.66 (±2.62)	1.82 (±2.03)	0.9926	5.51	16
22	14	1.000	5	0.200	12.63 (±4.11)	3.31 (±3.7)	0.7009	9.02	12
29	58	1.000	26	0.125	14.80 (±2.09)	4.40 (±2.07)	0.5381	21.7	23
31	16	1.000	4	0.167	14.97 (±1.66)	1.61 (±2.29)	0.5937	7.42	9
32	29	1.000	13	0.125	15.29 (±2.35)	3.74 (±2.4)	0.6517	14.22	17

NT: number of individuals counted in the codend; qNT: codend sampling ratio; NC: number of individuals counted in the codend cover; qNC: codend cover sampling ratio; L50: 50% retention length; SR: selection range (L75-L25); CI: confidence interval; DOF: degrees of freedom.

**Table 3:** Description and model ranking based on the full model (Eq 2) for Atlantic horse mackerel.

Model rank	AICc	Delta AICc	Akaike weight	Para-meter	Factor					
					a_0_, b_0_	a_1_, b_1_	a_2_, b_2_	a_3_, b_3_	a_4_, b_4_	a_5_, b_5_
1	93.69	0.00	0.5400	L50	25.91 (2.48)	-	-0.21 (0.05)	-2.94 (0.74)	-	-
				SR	1.72 (0.53)	1.67 (0.58)	-	-	-	-
2	95.50	1.80	0.2191	L50	20.52 (2.68)	1.82 (0.67)	-0.14 (0.05)	-1.98 (0.74)	-	-
				SR	2.83 (0.32)	-	-	-	-	-
3	96.27	2.57	0.1492	L50	12.34 (0.66)	2.52 (0.74)	-	-	-	-
				SR	2.95 (0.26)	-	-	-	-	-
4	97.48	3.78	0.0815	L50	23.84 (2.08)	-	-0.17 (0.04)	-2.39 (0.55)	-	-
				SR	2.91 (0.34)	-	-	-	-	-
5	101.62	7.93	0.0103	L50	14.13 (0.45)	-	-	-	-	-
				SR	1.83 (0.62)	1.67 (0.69)	-	-	-	-

Delta AICc: difference between the AICc value of two models: the model used and the one with the lowest AICc value. Values in brackets: standard error.

**Fig 2:**
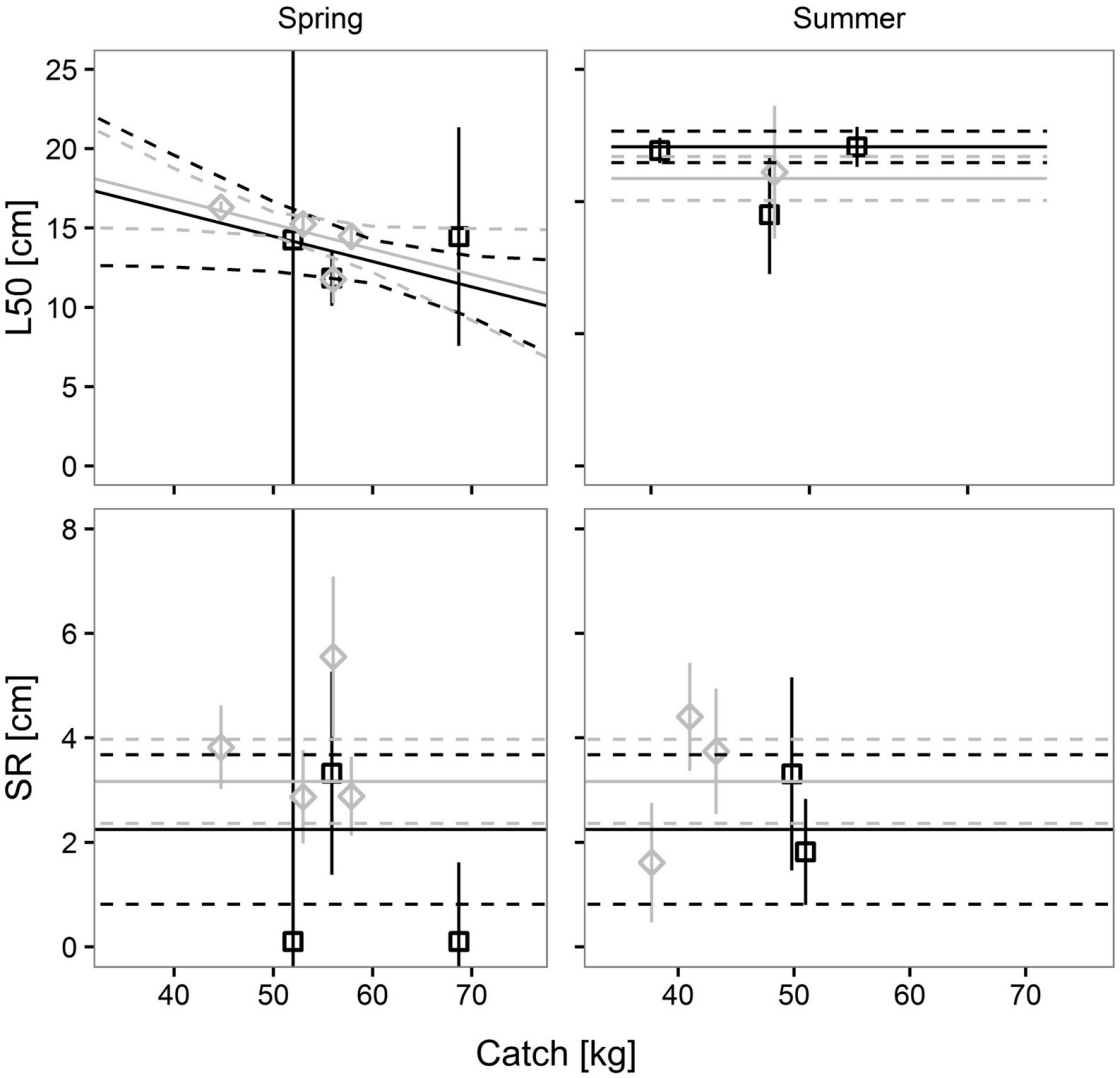
Prediction of Atlantic horse mackerel size selection parameters, L50 and SR, versus codend catch weight. Black solid and dashed lines represent the predicted mean values and 95% CI for the SM40 codend. Grey solid and dashed lines represent the predicted mean values and 95% CI for the DM50 codend. Squares and diamonds represent the results of each haul and their 95% CI for the SM40 and the DM50 codend, respectively.

Factor *a*_*1*_ was found in only 2 of the 5 models; their Akaike weights were respectively 0.2191 and 0.1492, meaning that the two models had some effect on *L50* prediction. As regards *SR*, factor *b*_*1*_ was found in two models, and its relatively high Akaike weight (0.5503) suggested a strong influence of codend design on predicted *SR* values. Factors *a*_*2*_ and *a*_*3*_ were found in 3 of the 5 models. Their negative sign indicates a reduction in predicted L50 values with the increase in codend catch weight, and smaller predicted *L50* values in summer compared with spring. Neither catch weight nor season affected the predicted *SR* values. Factors *a*_*4*_, *b*_*4*_ and *a*_*5*_, *b*_*5*_ were not found in the models. The predictive model for Atlantic horse mackerel agrees with the results of the individual hauls (Fig 2), demonstrating its ability to be used in predictions.

**Fig 3:**
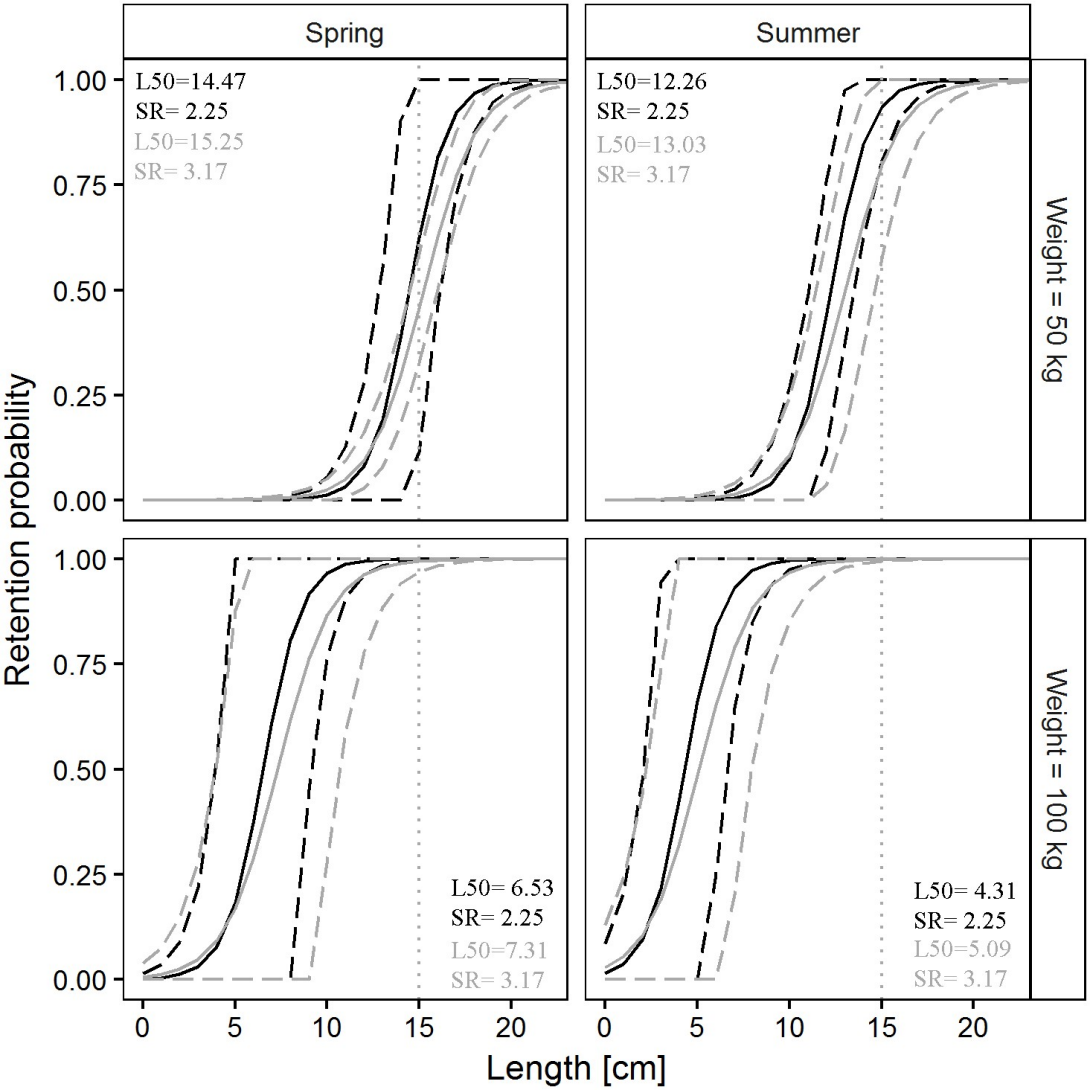
Differences in Atlantic horse mackerel retention probability between the SM40 (black) and the DM50 (grey) codend in four simulated scenarios. Dashed lines: 95% CI for the mean curve (solid line); dotted vertical line: MCRS for Atlantic horse mackerel (TL, 15 cm).

The pairwise comparisons of the size selection properties of the SM40 and the DM50 codend for Atlantic horse mackerel in four simulated scenarios are shown in Fig 3. The complete overlap of the 95% CI of the predicted selectivity curves for the SM40 and the DM50 codend indicates that there is no difference in selectivity between the codends in the four scenarios.

#### European hake

The predictive model for European hake was obtained from data from 16 hauls, for which it was possible to obtain a size selection curve (Table 4). Out of a total number of 955 individuals caught during the experiment, 615 were caught with the SM40 (412 length measured) and 340 with the DM50 codend (247 length measured). The logit curve described the experimental data in a satisfactory way, with p-values > 0.05 in all considered hauls (Table 4). The results of each haul were then used to establish the predictive model for *L50* and *SR*. Details of the 5 models that yielded AICc values within +10 of the model with the lowest value are reported in Table 5.

**Table 4:** Estimated selection parameters and fit statistics for European hake (16 hauls).

Haul	NT	qNT	NC	qNC	L50 [cm] (± CI)	SR [cm] (± CI)	p-value	Deviance	DOF
1	48	1.000	4	1.000	10.62 (± 3.64)	3.22 (± 2.91)	1.0000	6.42	30
3	52	1.000	2	1.000	9.25 (± 251.61)	0.10 (± 35.79)	1.0000	0.00	24
9	43	1.000	34	1.000	14.97 (± 0.75)	0.67 (± 0.89)	1.0000	2.14	32
10	40	1.000	9	1.000	14.39 (± 3.43)	2.73 (± 3.05)	1.0000	2.92	24
11	17	1.000	16	1.000	11.53(± 2836.8)	0.10 (± 297.4)	1.0000	0.00	17
12	47	1.000	19	0.500	13.78 (± 0.74)	0.10 (± 0.37)	1.0000	0.03	28
19	38	1.000	1	0.167	14.33(± 257.84)	0.10 (± 36.43)	1.0000	0.00	18
20	23	1.000	1	0.250	15.19 (± 1.80)	1.48 (± 3.21)	0.9947	3.11	12
21	37	1.000	9	0.200	13.70 (± 1.94)	3.49 (± 3.07)	0.9731	13.26	25
22	24	1.000	8	0.200	13.74 (± 2.51)	3.71 (± 3.63)	0.5397	15.78	17
23	10	1.000	6	0.100	13.66 (± 3.00)	2.09 (± 2.65)	0.8329	5.02	9
24	24	1.000	6	0.100	13.56 (± 3.00)	2.43 (± 2.73)	0.9536	9.25	18
29	46	1.000	5	0.125	11.08 (± 0.61)	0.57 (± 0.88)	1.0000	0.13	21
30	36	1.000	3	0.167	10.65 (± 0.80)	0.70 (± 0.88)	1.0000	3.13	20
31	21	1.000	3	0.167	13.05 (± 2.51)	3.43 (± 5.04)	0.9480	6.63	14
32	23	1.000	4	0.125	12.89 (± 1.48)	1.5 (± 2.12)	1.0000	1.17	12

NT: number of individuals counted in the codend; qNT: codend sampling ratio; NC: number of individuals counted in the codend cover; qNC: codend cover sampling ratio; L50: 50% retention length; SR: selection range (L75-L25); CI: confidence intervals; DOF: degrees of freedom.

**Table 5:** Description and model ranking based on the full model (Eq 2) for European hake.

Model rank	AICc	Delta AICc	Akaike weight	Para-meter	Factor					
					a_0_, b_0_	a_1_, b_1_	a_2_, b_2_	a_3_, b_3_	a_4_, b_4_	a_5_, b_5_
1	148.57	0.00	0.5321	L50	7.79 (2.13)	-	0.11 (0.04)	-	-	-
				SR	-5.75 (2.33)	3.58 (1.50)	0.11 (0.04)	2.84 (0.59)	-	-4.00 (1.54)
2	150.36	1.79	0.2171	L50	8.19 (2.10)	-	0.11 (0.04)	-	-	-
				SR	-4.78 (2.22)	-	0.1 (0.04)	2.50 (0.51)	-	-
3	150.87	2.30	0.1682	L50	15.18 (0.44)	-1.16 (0.44)	-	-2.22 (0.49)	-	-
				SR	1.37 (0.30)	-	-	-	-	-
4	153.72	5.15	0.0405	L50	14.00 (0.37)	-2.39 (0.57)	-	-	-	-
				SR	1.59 (0.36)	-	-	-	-	-
5	153.88	5.31	0.0373	L50	15.46 (0.54)	-	-	-3.23 (0.45)	-	-
				SR	1.35 (0.31)	-	-	-	-	-
6	158.03	9.47	0.0047	L50	8.38 (2.24)	-	0.1 (0.05)	-	-	-
				SR	1.33 (0.32)	-	-	-	-	-

Delta AICc: difference between the AICc value of two models: the model used and the one with the lowest AICc value. Values in brackets: standard error.

**Fig 4:**
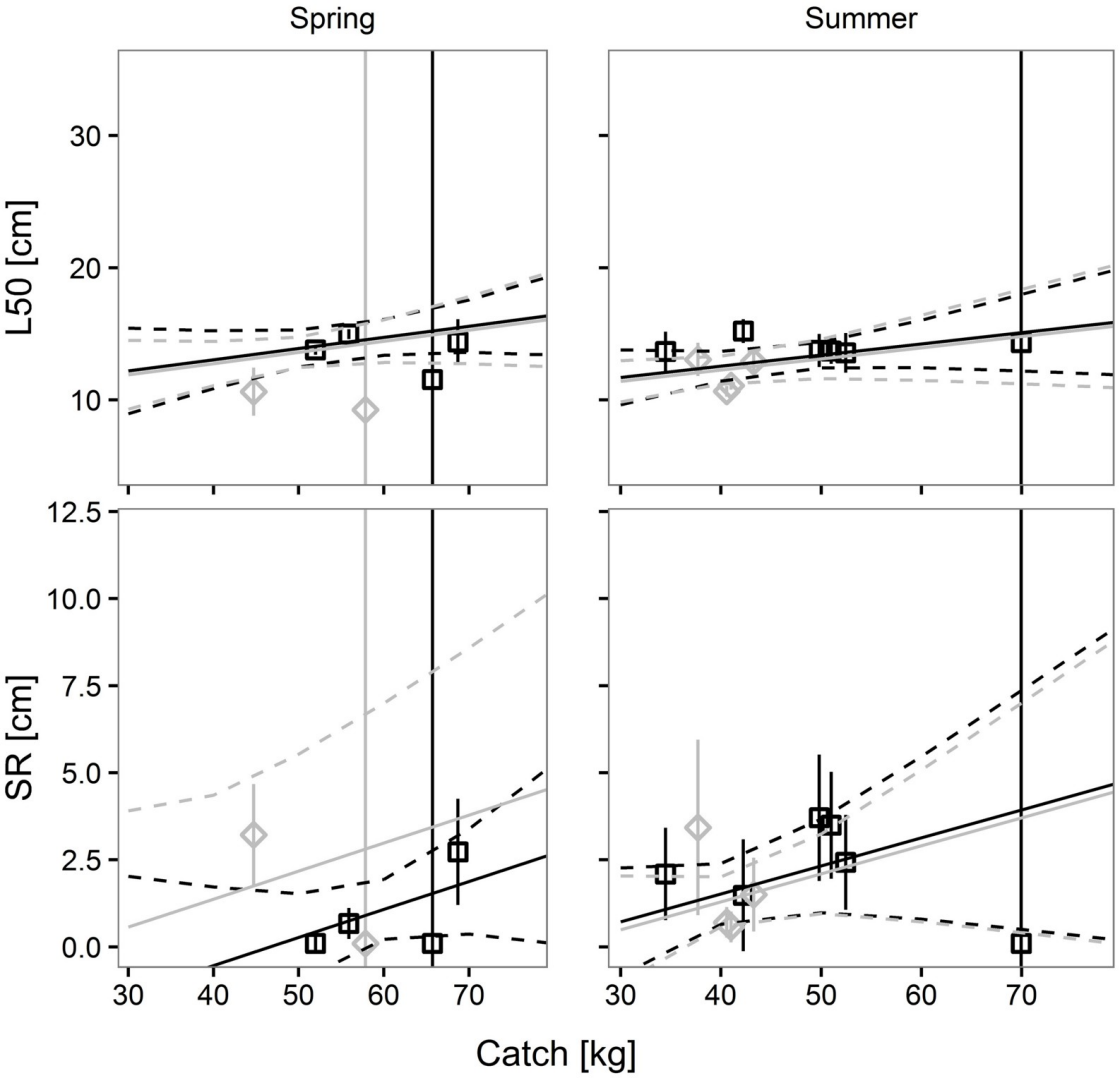
Prediction of European hake size selection parameters, *L50* and *SR*, versus codend catch weight. Black solid and dashed lines represent the predicted mean values and 95% CI for the SM40 codend. Grey solid and dashed lines represent the predicted mean values and 95% CI for the DM50 codend. Squares and diamonds represent the results of each haul and their 95% CI for the SM40 and the DM50 codend, respectively.

Factor *a*_*1*_ was found only in 2 of the 6 models; their relatively small Akaike weights, respectively 0.1682 and 0.0405, indicated that the two models had a very limited influence on *L50* prediction. As regards *SR*, factor *b*_*1*_ was found in one model; its relatively high Akaike weight (0.5321) suggested a strong influence of codend design on predicted *SR* values. In contrast, the two models including factors *a*_*2*_ and *b*_*2*_ displayed relatively high Akaike values (0.753 and 0.7492, respectively), which indicated a strong effect of codend catch weight on predicted L50 and SR values. The models containing factor *a*_*3*_ had different Akaike weights for *L50* and *SR* (respectively 0.2055 and 0.7942), suggesting that season influenced *SR* more than *L50*. The interaction between codend design and codend catch weight had no effect on *L50* and *SR* prediction, since none of the models included *a*_*4*_ or *b*_*4*_. In contrast, the interaction between codend design and season influenced only *SR* prediction. The predictive model for European hake agreed with the results of the single hauls (Fig 4), indicating its ability to be used in making predictions for this species.

**Fig 5:**
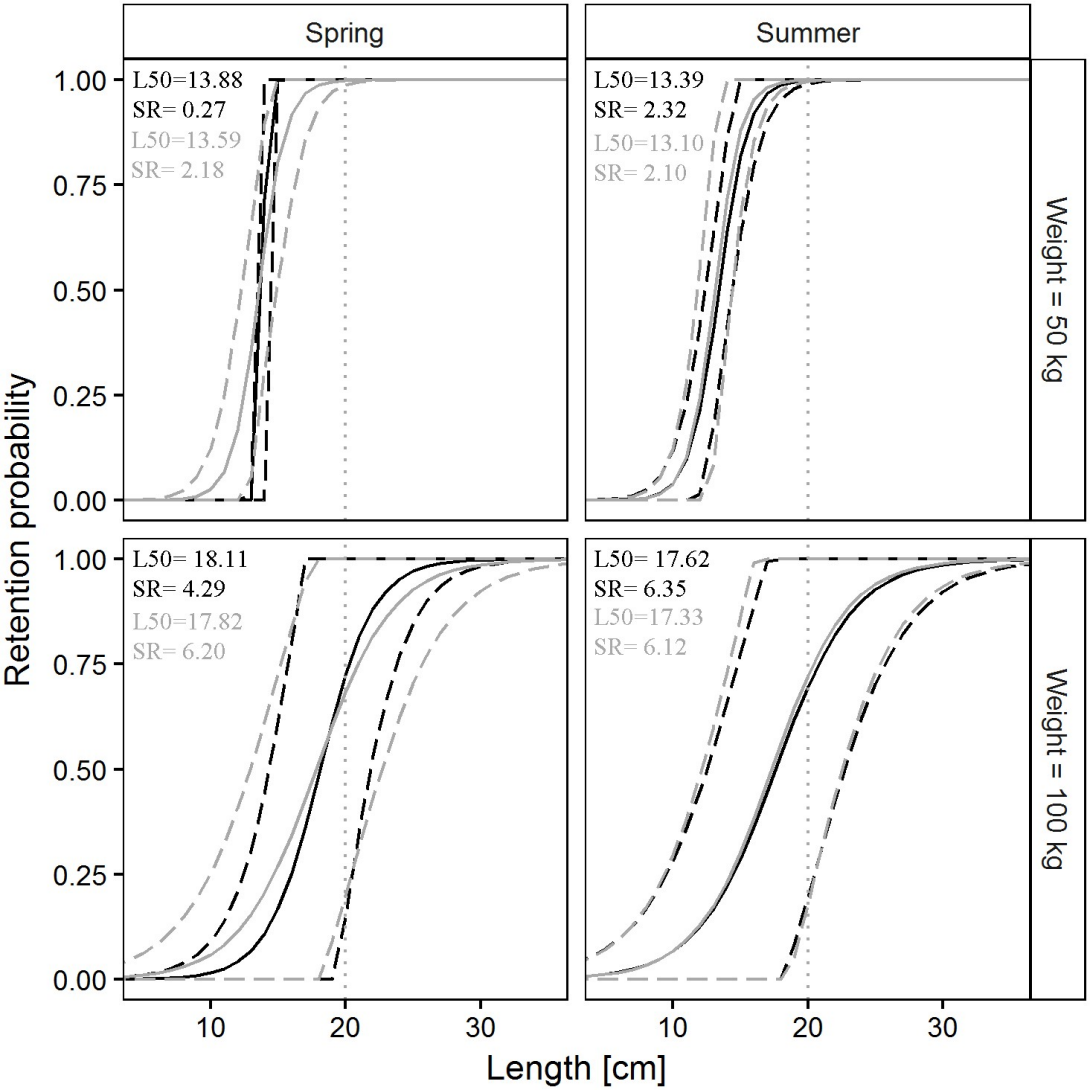
Differences in European hake retention probability between the SM40 (black) and the DM50 (grey) codend in four simulated scenarios. Dashed lines: 95% CI for the mean curve (solid line); dotted vertical line: MCRS for European hake (TL, 20 cm).

The pairwise comparisons of the predicted size selection curves for European hake are shown in Fig 5. The figure shows that there are no significant differences in selectivity between the two legal codends in the four simulated scenarios.

#### Norway lobster

The predictive model for Norway lobster was based on data from 12 hauls, for which it was possible to obtain a size selection curve. Altogether, 2735 individuals were caught with the SM40 codend (1055 length measured) and 1736 with the DM50 codend (685 length measured) (Table 6). Fitting of the logit curve (1) to the data from each considered haul consistently yielded p-values > 0.05, indicating that it was suitable to describe the experimental selection data of every haul (Table 6). The results of each haul were then used to establish the predictive model for *L50* and *SR*. A single model gave AICc values within +10 of the model with the lowest value (Table 7).

**Table 6:** Estimated selection parameters and fit statistics for Norway lobster (12 hauls).

Haul	NT	qNT	NC	qNC	L50 [cm] (± CI)	SR [cm] (± CI)	p-value	Deviance	DOF
5	143	0.400	5	0.500	17.54 (± 2.32)	2.36 (± 1.70)	0.8362	14.73	21
6	228	0.500	19	1.000	18.15 (± 1.84)	3.90 (± 1.72)	0.9992	9.61	27
7	83	0.388	9	1.000	0.10 (± 20.76)	18.75 (± 15.39)	0.3809	26.51	25
8	92	0.250	9	1.000	15.20 (± 4.25)	4.49 (± 2.97)	0.9999	5.83	23
28	95	0.333	2	0.250	22.22 (± 10.04)	3.39 (± 6.68)	1.0000	2.78	19
13	172	0.500	10	0.250	20.46 (± 1.10)	2.73 (± 1.76)	0.9978	10.73	27
14	117	0.250	2	0.250	15.48 (± 7.91)	5.39 (± 5.88)	0.9998	8.56	28
15	196	0.333	21	0.500	19.94 (± 1.43)	3.42 (± 1.56)	0.9955	11.67	27
16	155	0.500	44	1.000	20.40 (± 1.10)	3.96 (± 1.49)	0.9506	16.12	27
17	162	0.500	3	0.200	24.14 (± 3.03)	3.15 (± 3.33)	0.9994	7.65	24
19	96	0.250	2	0.167	19.01 (± 9.01)	5.08 (± 5.71)	0.9999	3.67	18
20	72	0.500	3	0.250	23.87 (± 1.91)	1.87 (± 2.12)	0.9999	3.63	19

NT: number of individuals counted in the codend; qNT: codend sampling ratio; NC: number of individuals counted in the codend cover; qNC: codend cover sampling ratio; L50: 50% retention length; SR: selection range (L75-L25); CI: confidence intervals; DOF: degrees of freedom.

**Table 7:** Description and model ranking based on the full model (Eq 2) for Norway lobster.

Model rank	AICc	Delta AICc	Akaike weight	Parameter	Factor					
					a_0_, b_0_	a_1_, b_1_	a_2_, b_2_	a_3_, b_3_	a_4_, b_4_	a_5_, b_5_
1	88.64	0.00	0.9789	L50	20.29 (0.33)	-2.45 (0.59)	-	3.85 (0.66)	-	-
				SR	3.12 (0.33)	-	-	-	-	-
2	96.31	7.68	0.0211	L50	19.17 (0.53)	-	-	4.68 (1.00)	-	-
				SR	3.01 (0.30)	-	-	-	-	-

Delta AICc: difference between the AICc value of two models: the model used and the one with the lowest AICc value. Values in brackets: standard error.

**Fig 6:**
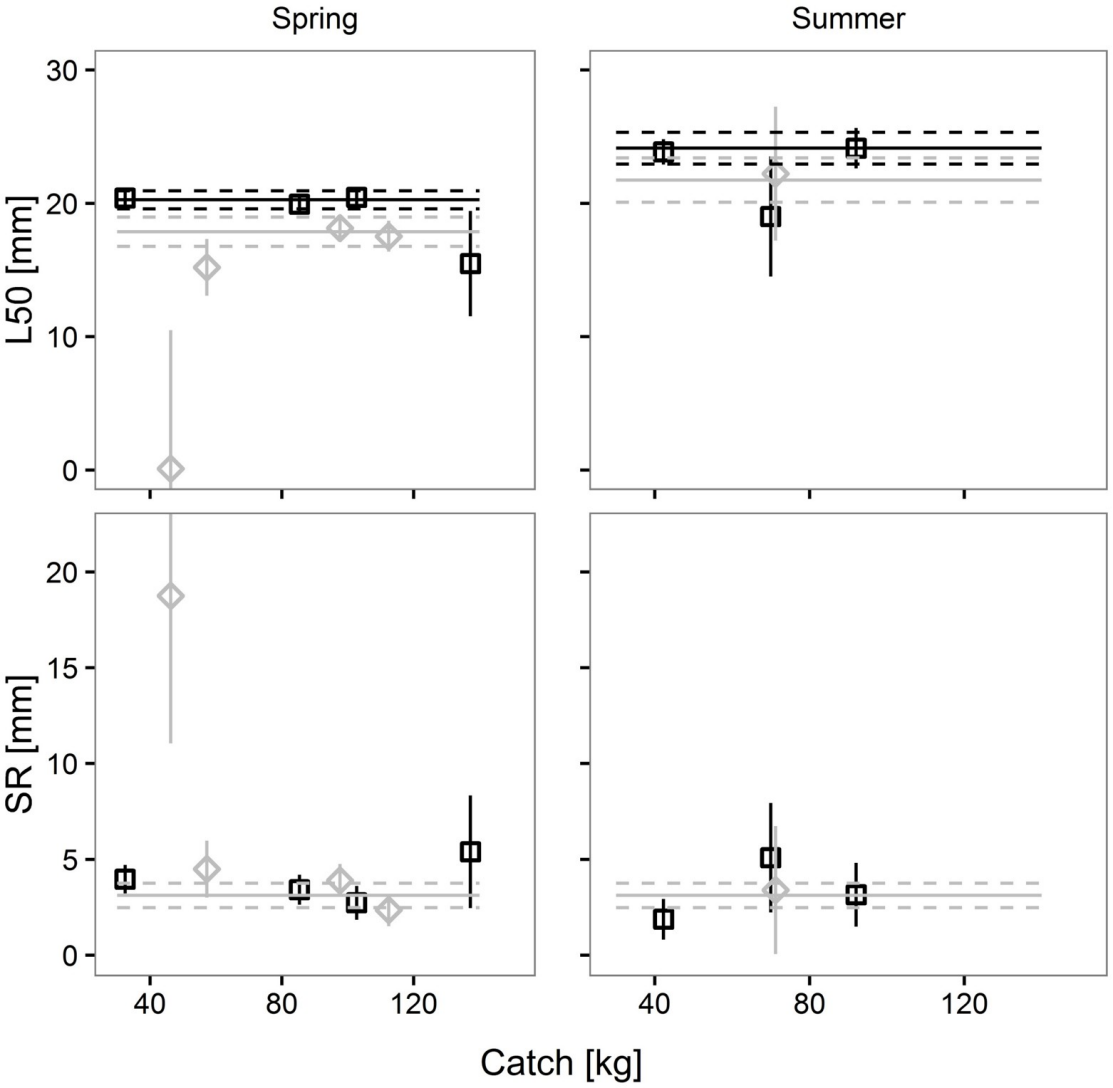
Prediction of Norway lobster size selectivity parameters, L50 and SR, versus codend catch weight. Black solid and dashed lines represent the predicted mean values and 95% CI for the SM40 codend. Grey solid and dashed lines represent the predicted mean values and 95% CI for the DM50 codend. Squares and diamonds represent the results of individual hauls and their 95% CI for the SM40 and the DM50 codend, respectively. The Black solid and dashed lines for SR are masked by the grey solid and dashed lines.

Codend design (Akaike weight = 0.9789) and season (sum of Akaike weights = 1) exerted a strong influence on predicted *L50* values. The negative value of factor *a*_*1*_ indicates that the switch from the SM40 to the DM50 codend resulted in lower *L50* values, whereas the positive value of *a*_*3*_ predicted higher *L50* values in summer than in spring. Neither codend design nor season affected *SR* prediction. Factors *a*_*2*_, *b*_*2*_, *a*_*4*_, *b*_*4*_ and *a*_*5*_, *b*_*5*_ were not found in the models. The model for Norway lobster (Table 7) reasonably agreed with the experimental results, supporting its value in making predictions for this species (Fig 6).

**Fig 7:**
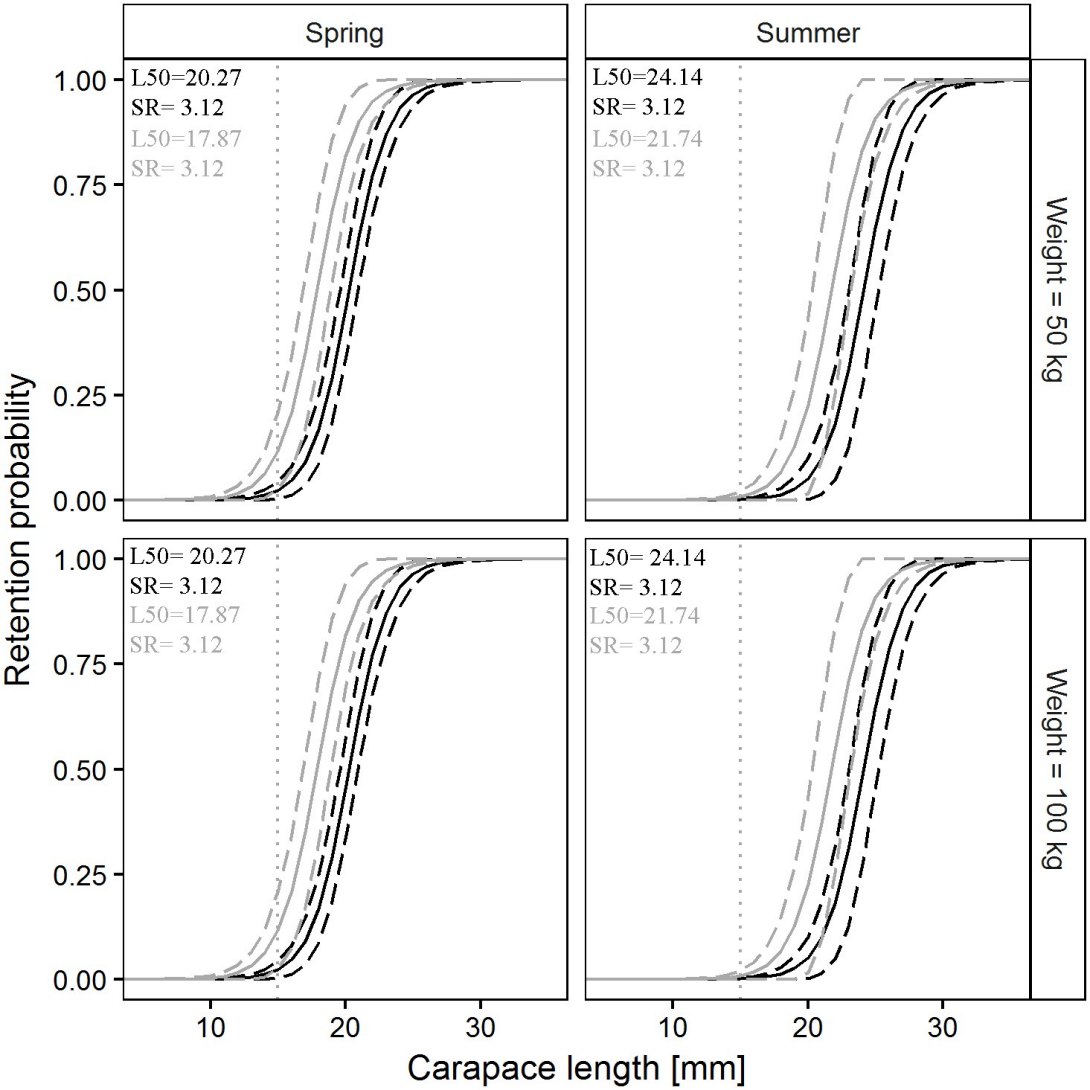
Differences in Norway lobster retention probability between the SM40 (black) and the DM50 (grey) codend in four simulated scenarios. Dashed lines: 95% CI for the mean curve (solid line); dotted vertical line: MCRS for Norway lobster (CL, 20 mm).

The pairwise comparisons of the predicted size selectivity curves for Norway lobster are shown in Fig 7. A difference in selectivity between the two codends was predicted only for spring, where the CI did not overlap.

#### Deep-water rose shrimp

Of a total number of 25,563 individuals of deep-water rose shrimp, 13,906 were caught with the SM40 gear (2959 length measured) and 11657 with the DM50 codend (2557 length measured). Data from 27 considered hauls (Table 8) for which it was possible to obtain a size selection curve (Table 9) were used to calculate the predictive model. In one case, the logit curve (1) failed to fit the experimental data (p–value < 0.05), but given the absence of systematic patterns in residuals the discrepancy between data and model is probably due to overdispersion in the data [24].

**Table 8:** Estimated selection parameters and fit statistics for the deep-water rose shrimp (27 hauls).

Haul	NT	qNT	NC	qNC	L50 [cm] (± CI)	SR [cm] (± CI)	p-value	Deviance	DOF
1	85	0.500	40	1.000	14.15 (± 2.42)	7.00 (± 2.76)	0.4218	17.49	17
2	140	0.399	22	1.000	9.21 (± 10.57)	9.77 (± 8.54)	0.5817	11.35	13
3	123	0.333	39	1.000	12.70 (± 3.06)	6.84 (± 2.74)	0.2342	23.07	19
4	129	0.250	59	1.000	14.17 (± 2.75)	6.68 (± 2.56)	0.3186	22.41	20
6	108	1.000	1	1.000	19.44 (± 7.78)	4.03 (± 4.77)	0.9250	7.24	14
7	95	1.000	1	1.000	6.57 (± 97.64)	11.73 (± 48.51)	0.9851	3.90	12
8	89	0.250	3	1.000	11.07 (± 23.17)	8.33 (± 11.47)	0.9314	7.80	15
9	135	1.000	52	1.000	17.11 (± 1.23)	3.36 (± 1.11)	0.5305	20.84	22
10	248	0.500	67	1.000	16.61 (± 1.02)	3.13 (± 0.93)	0.9220	11.82	20
11	208	0.500	24	0.250	17.39 (± 1.26)	3.65 (± 1.35)	0.9495	11.61	21
12	117	0.250	47	0.500	14.94 (± 2.22)	4.44 (± 1.69)	0.5361	13.86	15
13	208	0.250	9	0.250	21.89 (± 1.73)	2.09 (± 1.48)	0.9894	7.71	19
17	179	0.167	3	0.200	20.26 (± 2.38)	1.94 (± 1.78)	0.9983	4.28	16
18	147	0.200	1	0.167	18.38 (± 7.93)	3.27 (± 5.01)	0.9997	3.23	16
19	223	0.200	2	0.167	19.69 (± 5.71)	3.11 (± 3.73)	0.9974	4.10	15
20	199	1.000	2	0.250	15.33 (± 17.7)	7.65 (± 13.33)	0.9967	5.88	18
21	398	0.200	66	0.200	17.90 (± 0.64)	2.20 (± 0.58)	0.9917	10.58	24
22	240	0.100	38	0.200	16.03 (± 1.22)	3.28 (± 1.00)	0.7077	20.73	25
23	127	0.100	33	0.100	17.05 (± 1.1)	3.13 (± 1.09)	0.8095	15.26	21
24	160	0.125	26	0.100	14.91 (± 1.55)	5.29 (± 1.93)	0.3260	23.34	21
26	181	0.198	2	0.250	16.98 (± 11.72)	4.33 (± 6.48)	0.9985	3.72	15
27	146	0.167	4	0.125	16.59 (± 10.32)	6.88 (± 7.6)	0.9825	5.83	15
28	302	0.250	4	0.250	17.91 (± 6.4)	3.56 (± 3.89)	0.8611	10.11	16
29	193	0.167	50	0.125	18.58 (± 0.8)	2.74 (± 0.89)	0.5546	16.54	18
30	179	0.125	73	0.167	18.86 (± 1.1)	4.93 (± 1.38)	0.0530	32.42	21
31	192	0.167	40	0.167	18.59 (± 1.17)	3.79 (± 1.3)	0.6133	17.61	20
32	201	0.167	56	0.125	18.56 (± 1.23)	4.22 (± 1.52)	0.0294	29.59	17

NT: number of individuals counted in the codend; qNT: codend sampling ratio; NC: number of individuals counted in the codend cover; qNC: codend cover sampling ratio; L50: 50% retention length; SR: selection range (L75-L25); CI: confidence intervals; DOF: degrees of freedom.

**Table 9:** Description and model ranking based on the full model (Eq 2) for deep-water rose shrimp.

Model rank	AICc	Delta AICc	Akaike weight	Parameter	Factor					
					a_0_, b_0_	a_1_, b_1_	a_2_, b_2_	a_3_, b_3_	a_4_, b_4_	a_5_, b_5_
1	193.62	0.00	0.7146	L50	12.37 (1.21)	3.63 (1.74)	0.06 (0.02)	2.13 (0.73)	-0.07 (0.03)	-
				SR	3.76 (0.41)	1.63 (0.51)	-	-1.12 (0.51)	-	-
2	197.21	3.59	0.1187	L50	13.58 (1.14)	-	0.04 (0.02)	2.20 (0.82)	-	-
				SR	3.79 (0.39)	1.46 (0.32)	-	-1.12 (0.50)	-	-
3	197.44	3.82	0.1059	L50	12.94 (1.16)	3.99 (1.74)	0.07 (0.02)	0.96 (0.47)	-0.07 (0.03)	-
				SR	3.19 (0.33)	1.46 (0.54)	-	-	-	-
4	198.77	5.16	0.0543	L50	9.65 (1.74)	1.69 (0.52)	0.1 (0.02)	2.75 (0.84)	-	-
				SR	6.91 (1.10)	-	-0.04 (0.02)	-1.49 (0.57)	-	-
5	203.01	9.39	0.0065	L50	15.31 (0.97)	-	0.04 (0.02)	-	-	-
				SR	3.18 (0.29)	1.39 (0.31)	-	-	-	-

Delta AICc: difference between the AICc value of two models: the model used and the one with the lowest AICc value. Values in brackets: standard error.

The selection parameters of each haul were used to establish a predictive model. Details of the four models that gave AICc values within +10 of the model with the lowest value are reported in Table 9.

**Fig 8:**
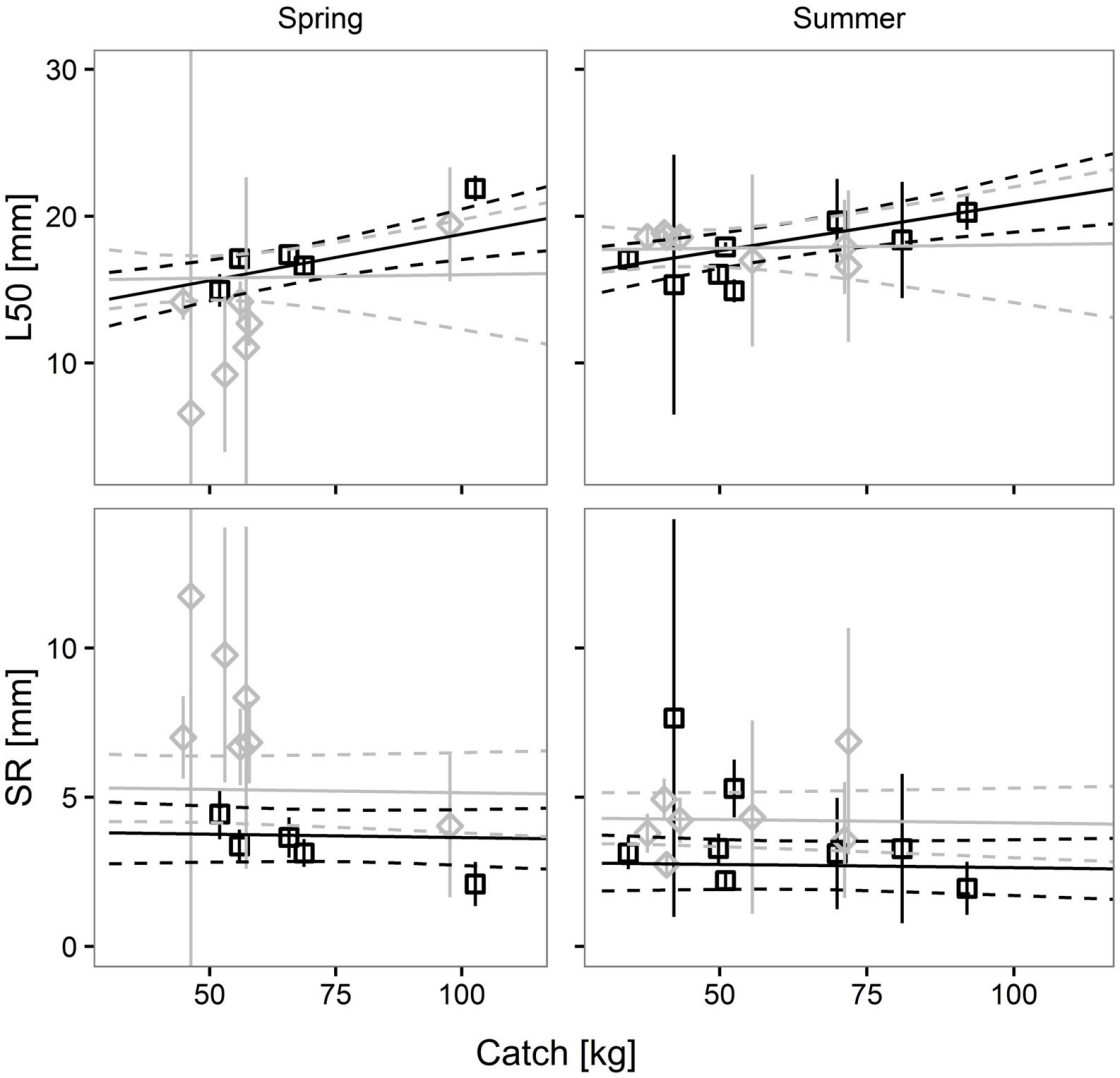
Prediction of deep-water rose shrimp size selectivity parameters, L50 and SR, versus codend catch weight. Black solid and dashed lines represent the predicted mean values and 95% CI for the SM40 codend. Grey solid and dashed lines represent the predicted mean values and 95% CI for the DM50 codend. Squares and diamonds represent the results of individual hauls and their 95% CI for the SM40 and the DM50 codend, respectively.

Factor *a*_*1*_ was found in 3 of the 5 models; the sum of their Akaike weights (0.8748) suggested a significant effect of switching from the SM40 to the DM50 codend. A similar situation was found for *SR*, where *b*_*1*_ appeared in 4 of the 5 models (sum of Akaike weights, 0.9457). Both *a*_*1*_ and *b*_*1*_ were positive, suggesting larger predicted *L50* and *SR* values for the DM50 codend. Factor *a*_*2*_ was found in all models, whereas *b*_*2*_ was included in a single model and showed a relatively low Akaike weight (0.0543). The positive value of *a*_*2*_ suggested an increase in predicted *L50* values with increasing codend catch weight, whereas the opposite was true for factor *b*_*2*_ and parameter *SR*. Factor *a*_*3*_ was found in 4 of the 5 and *b*_*3*_ appeared in 3 of the 5 models; the sums of their Akaike weights (0.9935 and 0.8876, respectively) suggested large seasonal differences in the selectivity of the two codends. The interaction between codend design and codend catch weight influenced predicted *L50* and *SR* values (sum of Akaike weights = 0.8205), whereas the absence of *a*_*5*_ and *b*_*5*_ suggested a lack of effect of the interaction of codend design and season on predicted *L50* and *SR* values. The predictive model for deep-water rose shrimp agrees with the results of the individual hauls (Fig 8), indicating its ability to make predictions for this species.

**Fig 9:**
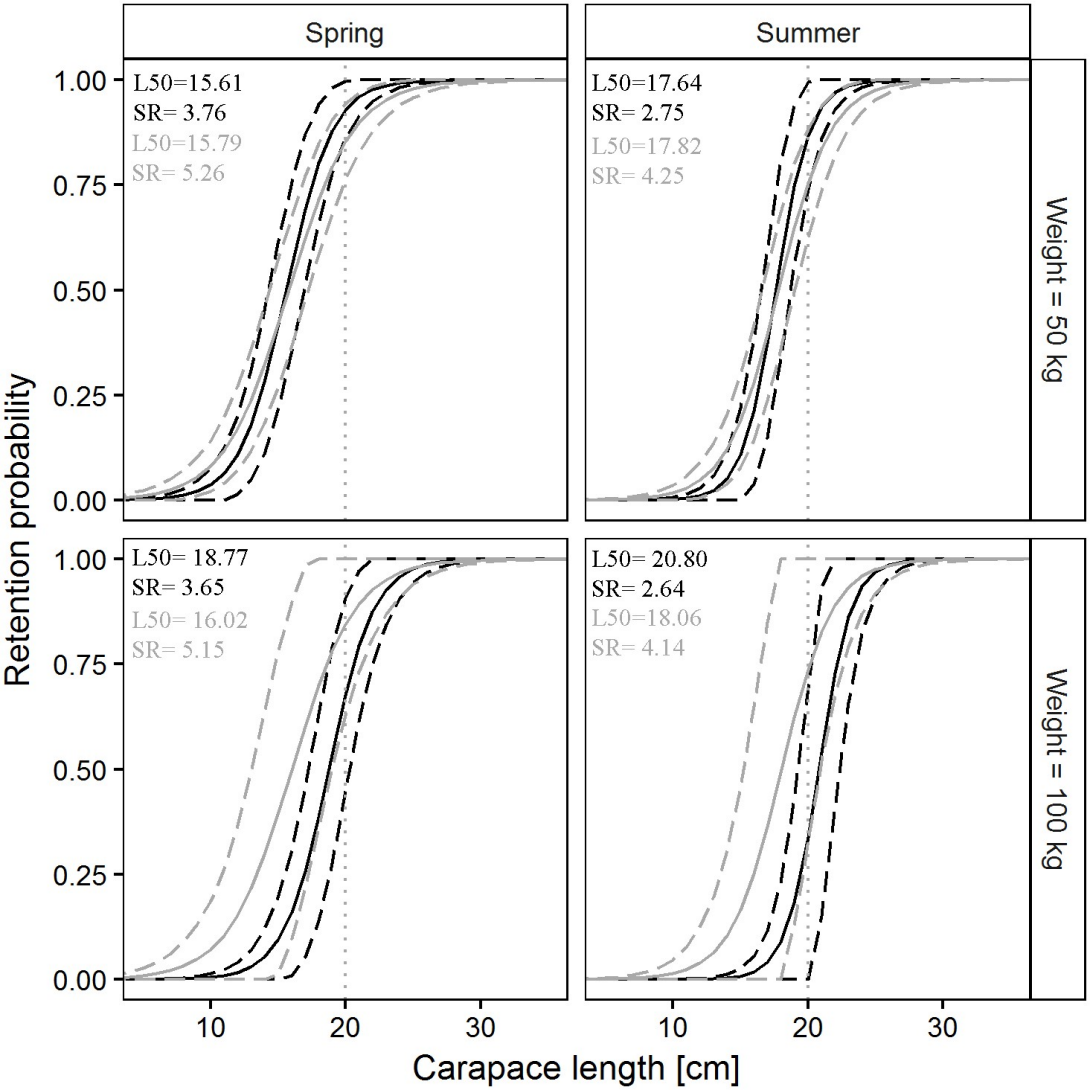
Differences in deep-water rose shrimp retention probability between the SM40 (black) and the DM50 (grey) codend in four simulated scenarios. Dashed lines: 95% CI for the mean curve (solid line); dotted vertical line: MCRS for deep-water rose shrimp (CL, 20 mm).

The pairwise comparisons of the predicted size selectivity curves of deep-water rose shrimp are shown in Fig 9. Examination of the figure indicates that there are no differences in selectivity between the two legal codends in any of the four simulated scenarios.

### Cross-species examination of the exploitation pattern indicators

**Fig 10:**
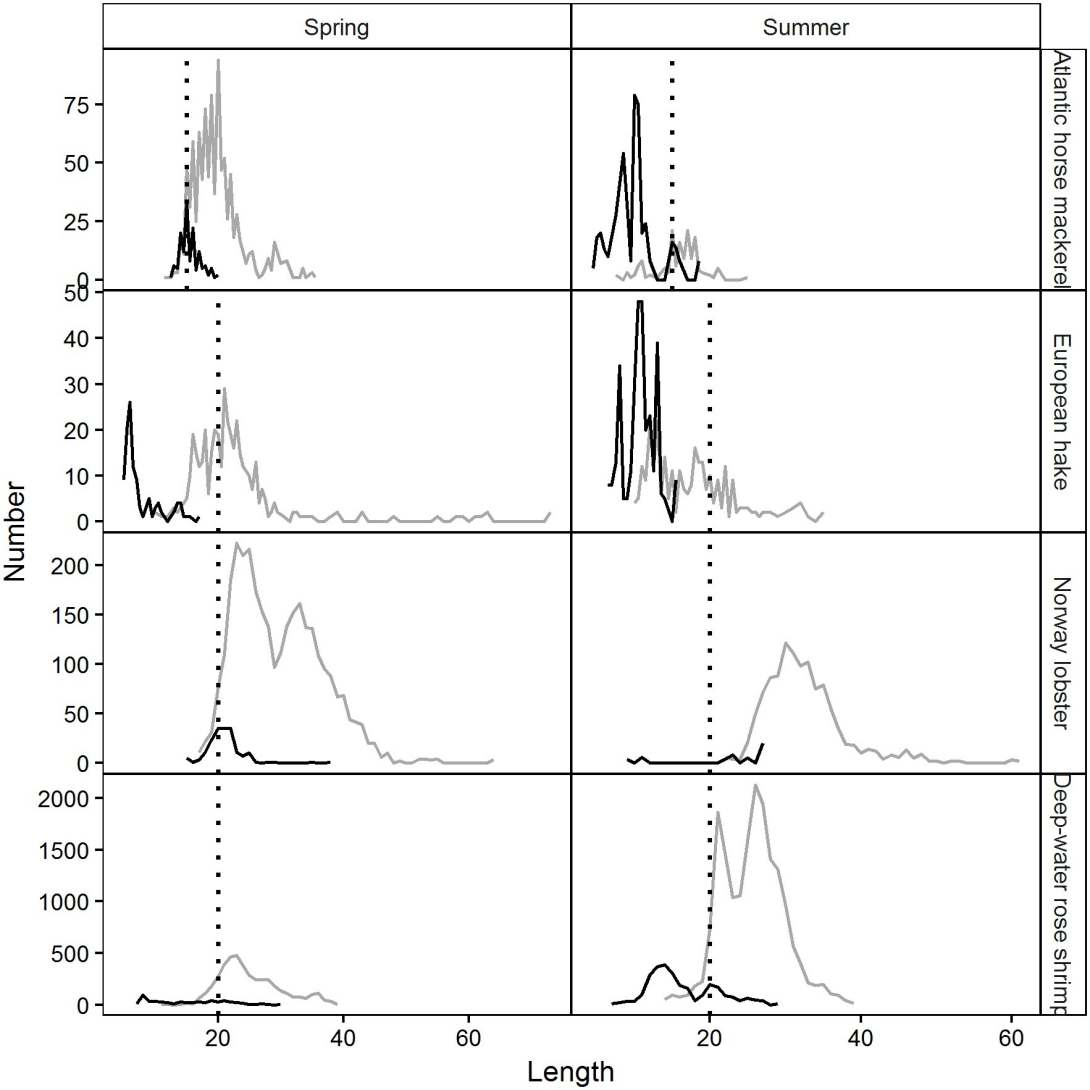
Length frequency distribution of analysed species retained by codend (grey) and cover (black) in spring and summer. Dotted vertical line: species Minimum Conservation Reference Size; TT: Atlantic horse mackerel, MM: European hake, NN: Norway lobster, PL: deep-water rose shrimp.

The pooled and raised seasonal size distributions of each species in each codend and cover are shown in Fig 10.

**Fig 11:**
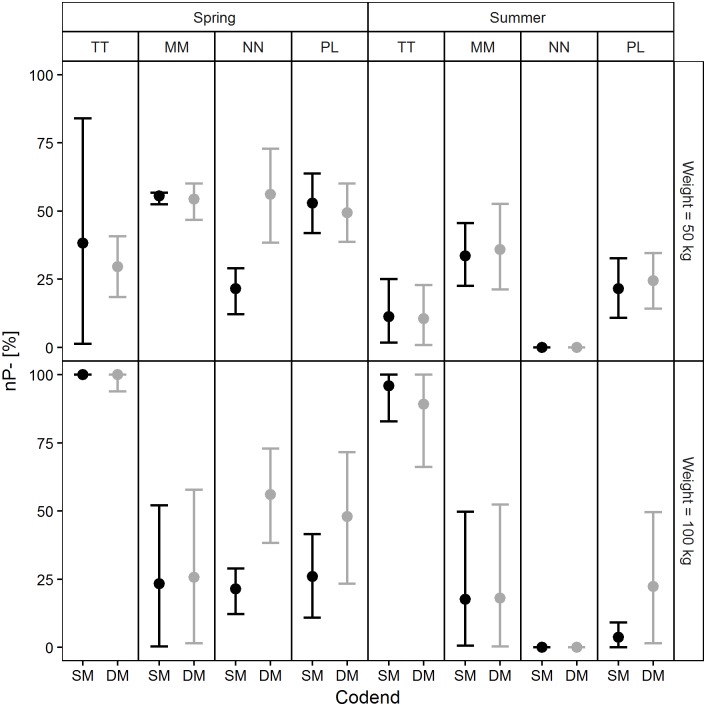
Percentage of retained individuals below the MCRS (*nP-* values). TT: Atlantic horse mackerel, MM: European hake, NN: Norway lobster, PL: deep-water rose shrimp.

**Fig 12:**
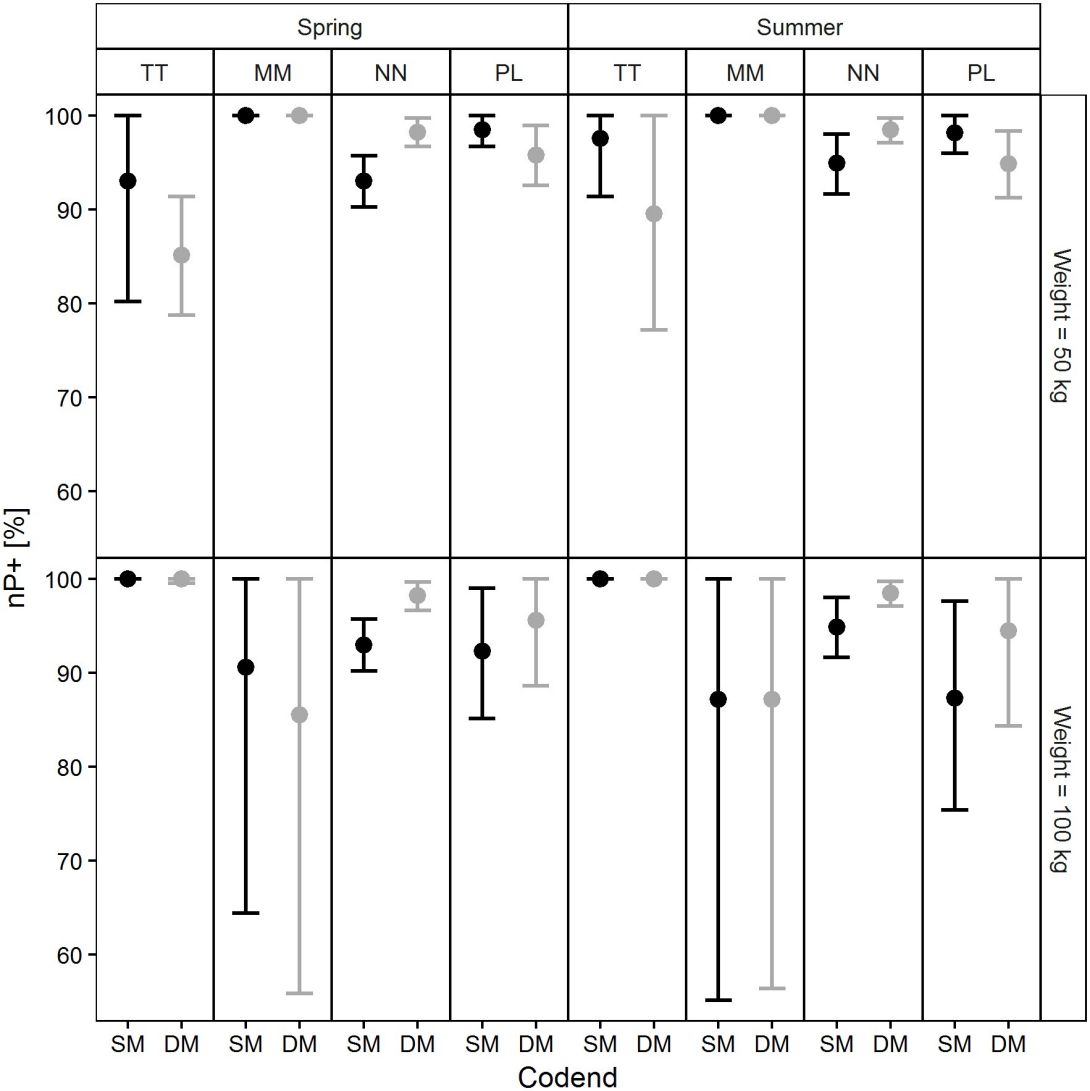
Percentage of retained individuals above the MCRS (*nP+* values). TT: Atlantic horse mackerel, MM: European hake, NN: Norway lobster, PL: deep-water rose shrimp.

**Fig 13:**
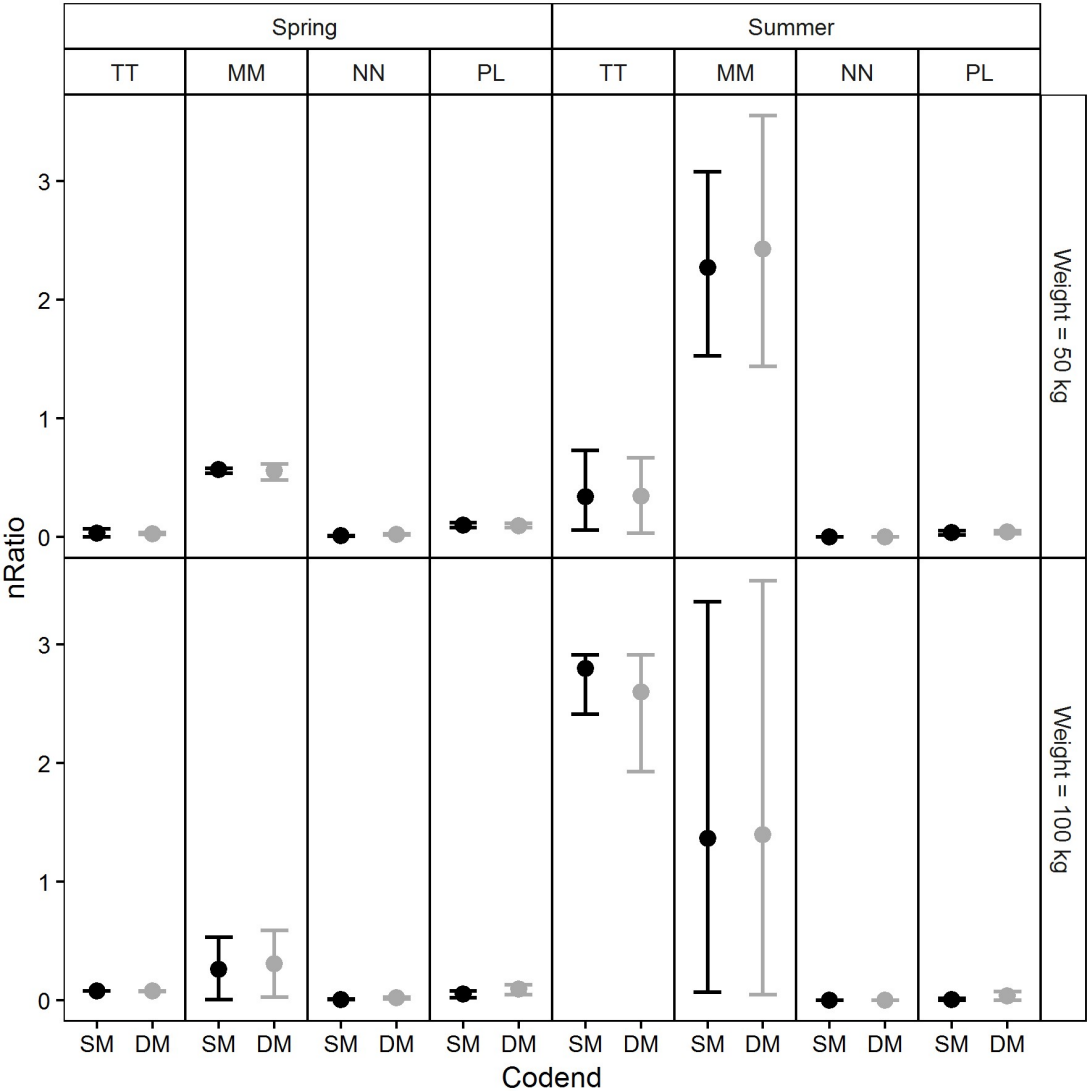
Number of retained individuals under the MCRS for each retained individual above the MCRS (*nRatio*). TT: Atlantic horse mackerel, MM: European hake, NN: Norway lobster, PL: deep-water rose shrimp.

**Fig 14:**
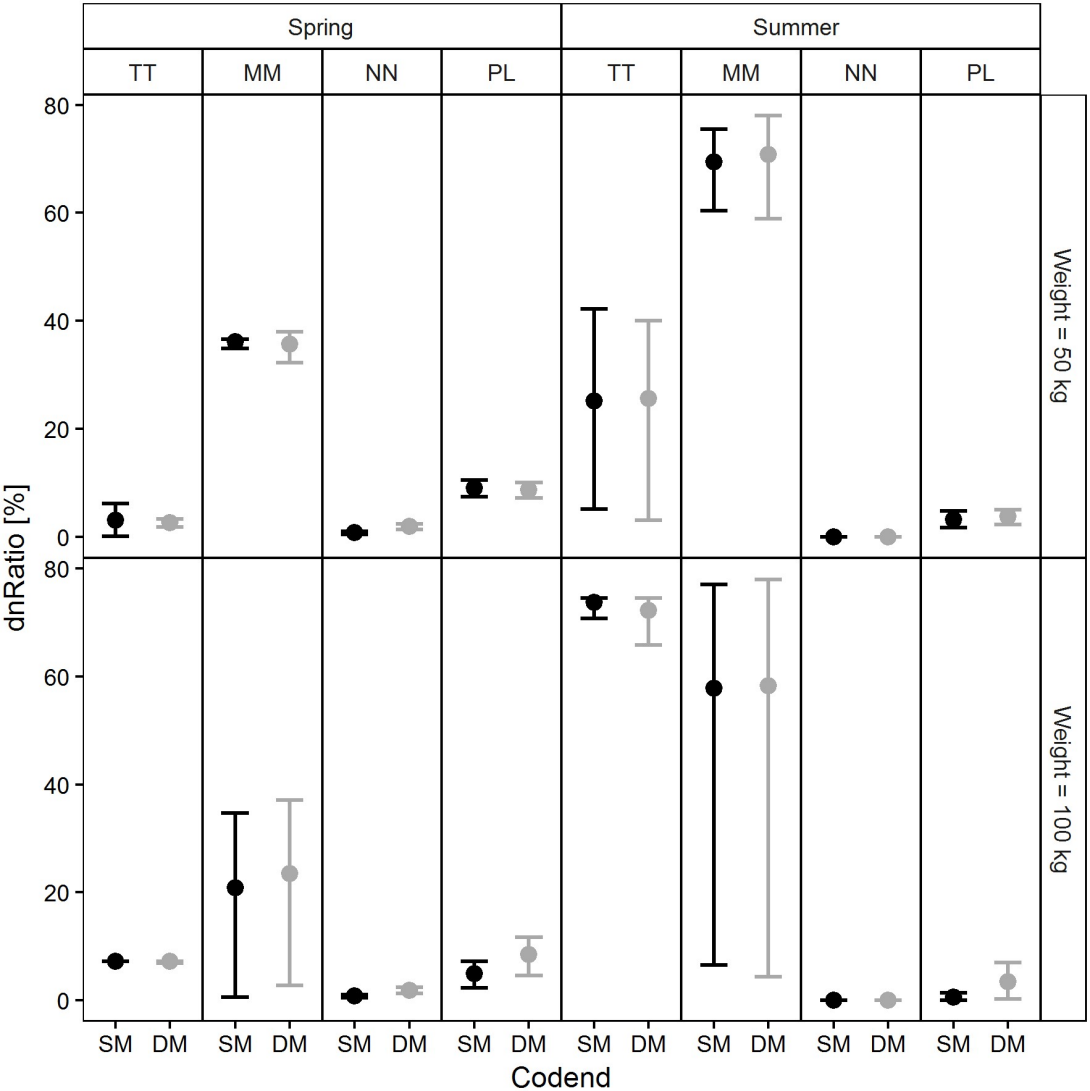
Percentage of individuals under the MCRS retained in the codend (*dnRatio*). TT: Atlantic horse mackerel, MM: European hake, NN: Norway lobster, PL: deep-water rose shrimp.

The exploitation pattern indicators calculated based on the population structures and size selectivity predictions made for each codend in four simulated scenarios are reported in Figs 11–14. Examination of the *nP-* indicator plot (Fig 11) shows that the two legal codends had a similar performance except for Norway lobster in spring, when the DM50 codend was predicted to retain significantly more individuals under the MCRS compared with the SM40 codend. The predictions were inconclusive for Atlantic horse mackerel (50 kg) in spring and for European hake (100 kg) in spring and summer, due to wide 95% CI (Fig 11).

Fig 12 shows that the two codends had a similar performance in terms of *nP+* values for all species except Norway lobster, where the DM50 codend retained significantly more individuals above the MCRS compared with the SM40 codend in spring. The predictions consistently yielded high *nP+* values for all species and all simulated scenarios, except for European hake (100 kg) due to very wide 95% CI (Fig 12).

Similar performances of the two codends were also found for the *nRatio* (Fig 13). The values of this indicator were consistently lower than 1, which means that for each individual under the MCRS the codends retained several individuals above the MCRS. The only exception was Atlantic horse mackerel; in this case the model predicted that several individuals under the MCRS would be caught for each individual above the MCRS retained in the codend (Fig 13). For European hake the results for summer were inconclusive due to wide 95% CI.

As regards the *dnRatio* (discard ratio), the only significant difference between the codends was predicted for Norway lobster in spring (Fig 14). The highest values were predicted for European hake in all four scenarios (although they were inconclusive in summer for the larger codend catch weight) and for Atlantic horse mackerel in summer.

## Discussion

The aim of this study was to establish predictive models enabling comparison of the size selectivity of the two legal codends—a 40 mm SM and a 50 mm DM codend—for four major commercial demersal species in Mediterranean bottom trawl fisheries: Atlantic horse mackerel, European hake, Norway lobster, and deep-water rose shrimp. The study was devised to help EU fisheries managers examine the ship-owners’ requests to use a 50 mm DM rather than a 40 mm SM codend. The models take into account the potential effect of codend catch size and fishing season. All four species have an MCRS defined by Council Regulation (EC) No. 1967/2006 and are therefore subject to the landing obligation.

The study documented a significant difference in size selection only for Norway lobster in spring, with a slightly better performance of the SM40 codend. The L50 values predicted for the SM40 codend in spring are significantly lower than those reported in the same season by [41] (27.4 mm), whereas the L50 values predicted in summer are in line with the autumn data reported both by the same researchers (24.5 mm) and by [7] (24.05 mm). As regards the slightly lower values reported by [5] (19.1 mm), the lack of 95% CI in their data prevents determining whether the difference is significant. The predictions regarding the DM50 codend can be compared only with the data reported by [42] and [43]. The predictions for spring are significantly lower than those reported by [42] in the same season (23.1 mm), whereas the L50 values of the two studies in summer are similar. As regards the paper by [43], analysis of the CI of their data highlighted a similarity between our spring predictions and their spring data for a 47 mm DM codend (20.06 mm) and a significant difference compared with their 51.7 DM codend (20.53 mm).

The L50 values predicted for the DM50 codend, for Atlantic horse mackerel were significantly different from those reported by [44] in the Aegean Sea (15.6 cm), except for spring at lower catch weights. The same is observed for the SM40 codend when compared with the results reported by [45] (15.9 cm).

The selectivity of the two codends for European hake was not significantly different (Table 5, Fig 5). The L50 predictions for the SM40 codend in spring do not differ significantly from the values reported by [6] (15.4 mm), while those for summer are in line with the L50 values reported by [45] (14.4 mm) and [5] (14.17 mm), although those studies were conducted in autumn and summer-autumn, respectively. Moreover, our SM40 predictions for the higher catch weight (100 kg) in summer are not significantly different from the data reported by [46] (15.2 mm) and [6] (15.3 mm) in autumn. Our predictions for the DM50 codend in summer do not differ significantly from those obtained by [44] (11.4 mm) in autumn.

As regards deep-water rose shrimp, the two codends did no exhibit significant differences in size selectivity (Fig 9 and Table 9). Our L50 predictions for the SM40 gear in spring are significantly lower than those reported by [41] (20.8 mm), whereas those for summer, for the higher catch weight, are not significantly different from the autumn data reported by the same researchers (20.3 mm). The spring SM40 predictions for the higher catch weight are not significantly different from the spring results reported by [47] (18.2 mm), but are different from those reported by [5] (14.9 mm) and [48] (16.29 mm); however, since these studies do not report the uncertainty around their estimates, it is impossible to determine whether the difference is significant. The predictions made for the DM50 codend are in line with the results reported by [48] for a 48 mm DM codend (16.61 mm).

Since the size selection properties of the two legal codends were compared in identical simulated controlled conditions, the predictions were limited to low catch rates, which are probably associated with the specific tow duration used in the study. The catch rates in the area vary considerably; however, since data were collected only for the lower bound of the catch rate, the prediction was possible only for low catch sizes. This entails that extrapolation of the results to the higher catch rates that can occur during commercial fishing is not necessarily feasible. Some caution is required when using the models for predictions across species for different conditions because some species were not captured across the tested levels for the three variables being studied. For example, hake and horse mackerel were entirely captured in hauls where codend catch size was well below 100 kg, whereas Nephrops was only captured in one summer haul with the DM50 codend.

Notably, since the data regard only spring and summer they do not allow to draw conclusions for autumn and winter catches, because seasonal factors such as sea state [49] and fish condition [50] may affect size selectivity. However, such effects are unreported for the species investigated in this study and we can only speculate about their potential effect.

Since both codend designs investigated caught a considerable amount of immature individuals of all four species, the present study demonstrates that the size selection properties of Mediterranean bottom trawls need further improvement. A critical issue related to the improvement of codend selectivity, is that fish escaping from codend meshes survive. While this assumption has been tested for species such as cod, saithe and haddock [51], those included in this study have not, to the best of our knowledge, been investigated in this type of studies in the range of small mesh sizes used in Mediterranean demersal fisheries.

In conclusion, the present data document different size selection properties only for Norway lobster, with a slightly better performance of the SM40 codend. Since the species is commercially valuable in the Mediterranean, our data may help fisheries managers reject requests by EU ship-owners to switch to the DM50 gear in fisheries where Norway lobster is the main target species.
